# Anomalous Magnetic Anisotropy Behaviour in Co-Rich and Fe-Rich Glass-Coated Microwires under Applied Stress

**DOI:** 10.3390/s23198068

**Published:** 2023-09-25

**Authors:** Alfonso García-Gómez, Juan María Blanco, Paula Corte-León, Mihail Ipatov, Álvaro González, Julián González, Arcady Zhukov, Valentina Zhukova

**Affiliations:** 1 Department of Polymers and Advanced Materials, University of Basque Country, UPV/EHU, 20018 San Sebastian, Spain; juanmaria.blanco@ehu.eus (J.M.B.); paula.corte@ehu.eus (P.C.-L.); mihail.ipatov@ehu.es (M.I.); alvaro.gonzalezv@ehu.eus (Á.G.); julianmaria.gonzalez@ehu.eus (J.G.); arkadi.joukov@ehu.eus (A.Z.); 2 Department of Applied Physics, Engineering School of Gipuzkoa, University of Basque Country, UPV/EHU, 20018 San Sebastian, Spain; 3 EHU Quantum Center, University of the Basque Country, UPV/EHU, 20018 San Sebastian, Spain; 4 IKERBASQUE, Basque Foundation for Science, 48011 Bilbao, Spain

**Keywords:** soft magnetic materials, magnetic anisotropy, amorphous microwires, magnetic sensors

## Abstract

In this article, we study the effect of annealing temperature and applied stress on the magnetic properties of ${\mathrm{Fe}}_{71.80}B_{13.27}{\mathrm{Si}}_{11.02}{\mathrm{Nb}}_{2.99}{\mathrm{Ni}}_{0.92}$ and ${\mathrm{Co}}_{65.34}{\mathrm{Si}}_{12.00}B_{10.20}{\mathrm{Cr}}_{8.48}{\mathrm{Fe}}_{3.90}{\mathrm{Mo}}_{0.08}$ microwires. An anomalous behavior of the coercive field is observed while applying stress, indicating nontrivial changes in the microwire magnetic anisotropy. The effect of applied stimuli on the magnetic anisotropy and magnetostriction constant in both microwires is also discussed.

## 1. Introduction

Studies of glass-coated magnetic microwires fabricated using the Taylor–Ulitovsky [1,2,3] technique, known since the 1960s, has attracted much attention due to their cheap fabrication costs and excellent soft magnetic properties, which are quite suitable for sensor fabrication within an industry 4.0 environment. These properties include the appearance of the giant magnetoimpedance effect (GMI), high magnetic field sensibility, magnetic bistability suitable for robust magnetic memory and a strong dependence of magnetic properties to applied stress. The insulating, biocompatible and flexible glass coating allows extending the applications of such glass-coated microwires toward biomedicine, electronic surveillance or smart composites [1].

The magnetic properties of this microwire family are strongly related to the stress distribution arising in the metallic nucleus during the preparation process, which is associated mostly with the presence of the glass coating and the rapid solidification of the composite microwires [1,4]. These interactions along with the applied stresses and relaxations generated by annealing have been proven to significantly affect the domain structure, magnetic anisotropy and magnetostriction coefficient, $\lambda_{s}$, of glass-coated microwires.

The cheap fabrication costs, low dimensions and high sensibility to external stresses put glass-coated microwires in a privileged spot for their use in structural health monitorization devices. This has been explored in several works [5,6,7] by embedding or attaching microwires on different structures in order to estimate the accumulated stress or applied pressure. In a previous work [7,8], we explored the use of Fe-rich and Co-rich microwires as strain sensors in concrete structures working at ambient temperature, and an interpolation function for device calibration was proposed.

In this work, we study the effect of temperature annealing on $\lambda_{s}$ and $H_{c}$ in the Co-rich ($\lambda_{s}∼0$) and Fe-rich ($\lambda_{s}>0$) [1,9] microwires previously studied in [8]. This is performed by measuring the coercive field, $H_{c}$, while applying longitudinal stress and estimating $\lambda_{s}$ using the small-angle magnetization rotation method (SAMR) on several samples previously annealed at different temperatures.

## 2. Materials and Methods

Both microwires were fabricated using the Taylor–Ulitovsky method [1,4]. The process starts with the precursor alloy ingot inside a glass tube, with both of them surrounded by an inducting coil. By passing an AC current through it, the inducting coil heats both the glass and alloy to melting temperature. Then, a glass capillary is pulled out of the glass and caught by a rotating pick-up spool; while the microwire is being pulled, a high quenching rate is obtained by using water as a coolant, obtaining an amorphous structure in both the metallic core and glass coating. The microwire continues drawing until the entire alloy ingot is consumed, forming a continuous microwire with a total length of several kilometers.

The atomic composition in atomic percentage (at %) and the diameters of the microwires studied in this work were the following: ${\mathrm{Fe}}_{71.80}B_{13.27}{\mathrm{Si}}_{11.02}{\mathrm{Nb}}_{2.99}{\mathrm{Ni}}_{0.92}$ (d=15.9 μm and D=24.5 μm) and ${\mathrm{Co}}_{65.34}{\mathrm{Si}}_{12.00}B_{10.20}{\mathrm{Cr}}_{8.48}{\mathrm{Fe}}_{3.90}{\mathrm{Mo}}_{0.08}$ (d=17.5 μm and D=22.2 μm), where *d* corresponds to the diameter of the metallic core and *D* corresponds to the diameter of the whole microwire. Samples of both microwires were annealed in a conventional furnace for one hour at temperatures ranging from 80 °C to 400 °C. The reasons for doping FeBSi microwires with Ni and Nb are that such doping allows not only enhancing glass-forming ability and obtaining large-diameter microwires but also improving their mechanical properties [10,11].

The hysteresis loops were measured using the fluxmetric method, as described in [12], while applying tensile stress to the microwire. The microwire’s magnetization was obtained by using both a compensation coil and a pick-up coil of *N* turns that eliminate parasitic signals that could interfere in the measurement of the hysteresis cycles. This combination of coils measures the induced electromotive force, ε, due to the change in the magnetic flux generated by the magnetic domain reversal inside the magnetic core, as described in (1).

(1) $$\varepsilon =-N\frac{d\phi }{dt}$$

(2) $$M=\frac{1}{N\mu_{0}A_{s}}\int \varepsilon dt$$

The magnetization of the sample and hysteresis loop is then obtained by integrating ε over time using Equation (2), where $A_{s}$ corresponds to the metallic cross-section of the microwire and $\mu_{0}$ is the vacuum magnetic permeability.

Applied stress on the metallic core of the microwire, $\sigma_{m}$, can be estimated using Equation (3) [13].

(3) $$\sigma_{m}=\frac{K\cdot T}{K\cdot S_{m}+S_{gl}}$$

where $S_{m}$ and $S_{gl}$ correspond to the metallic core and glass cross-sections, respectively, *T* is the applied mechanical load and $K=E_{m}/E_{gl}$, where $E_{m}$ is the metallic and $E_{gl}$ is the glass Young’s moduli. The Young’s moduli quotient was fixed to K∼2, which is in agreement with the values given in [13,14].

The magnetostriction constant, $\lambda_{s}$, was measured using the small-angle magnetization rotation method SAMR [15]. This method is based on the rapid rotation of the microwire’s magnetization around the easy axis under applied stress while passing through a low AC current. Initially, this method was developed and used for amorphous materials with a negative magnetostriction constant. However, recently, this method has also been extended for magnetic microwires with positive $\lambda_{s}$ with rectangular hysteresis loops [16].

**Figure 1:**
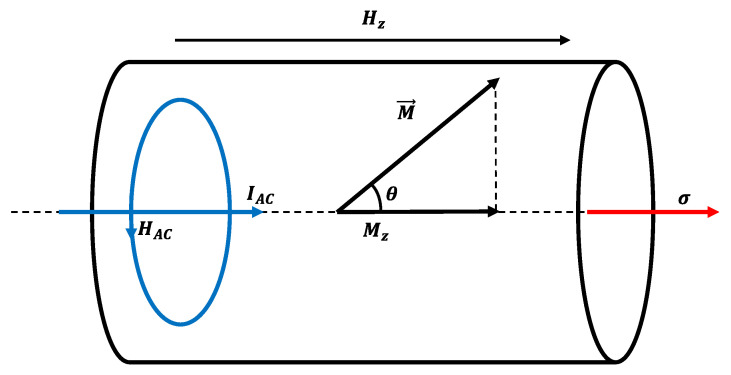
Scheme of the SAMR magnetic fields and moments inside the glass-coated microwire.

In our experimental set-up, the microwire was suspended with an attached mass that generates applied stress, $\sigma_{m}$, while inside the excitation and pick-up coil. The excitation coil generates a static magnetic field, $H_{z}$, that saturates the magnetization along the easy axis, $M_{z}$. Then, an alternating current, *I*, in the mA range and a driving frequency, f=10 kHz, is passed through the microwire. This current generates a circular oscillating magnetic field, $H_{AC}$, that leads to periodic deviations in the magnetization vector, $\vec{M}$, from the *z*-axis with an angle, θ, that oscillates with the second harmonic of the excitation current, 2f, as shown in Figure 1. The oscillation of the magnetization generates a periodic change in the easy axis magnetization, $M_{z}$, which produces an electromotive force that can be measured by the pick-up coil.

The value of the electromotive force (EMF) during the reversible magnetization oscillation can be fixed by changing the axial field, $H_{z}$. This way, $\lambda_{s}$ is obtained from the saturation magnetization, $M_{s}$, and the slope of the $H_{z}$ dependence of σ while keeping the EMF constant is given by the following expression:

(4) $$\lambda_{s}=\frac{\mu_{0}M_{s}}{3}\frac{dH_{z}}{d\sigma }$$

Structure of as-prepared and annealed samples were checked by X-ray diffraction (XRD) employing a BRUKER (D8 Advance) X-ray diffractometer with Cu K (λ=1.54 Å) radiation.

## 3. Results

**Figure 2:**
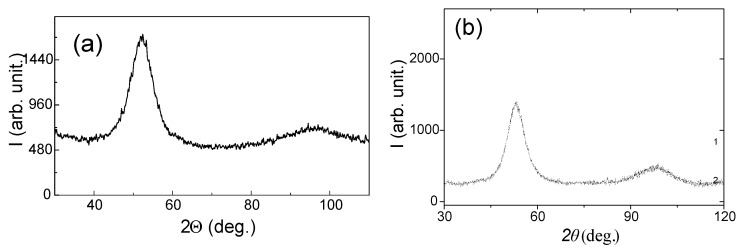
XRD of as-prepared microwire samples with the following composition: (**a**) ${\mathrm{Fe}}_{71.80}B_{13.27}{\mathrm{Si}}_{11.02}{\mathrm{Nb}}_{2.99}{\mathrm{Ni}}_{0.92}$, (**b**) ${\mathrm{Co}}_{65.34}{\mathrm{Si}}_{12.00}B_{10.20}{\mathrm{Cr}}_{8.48}{\mathrm{Fe}}_{3.90}{\mathrm{Mo}}_{0.08}$.

All as-prepared samples presented X-ray diffraction (XRD) patterns with a broad halo typical for completely amorphous materials, as shown in Figure 2. Similar XRD patterns were observed for the samples annealed over the whole range of $T_{ann}$ employed by us.

**Figure 3:**
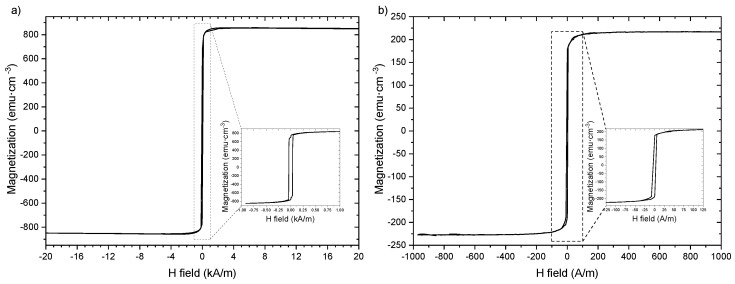
Hysteresis loops at high fields for the as-prepared microwires: (**a**) ${\mathrm{Fe}}_{71.80}B_{13.27}{\mathrm{Si}}_{11.02}{\mathrm{Nb}}_{2.99}{\mathrm{Ni}}_{0.92}$ microwire. (**b**) ${\mathrm{Co}}_{65.34}{\mathrm{Si}}_{12.00}B_{10.20}{\mathrm{Cr}}_{8.48}{\mathrm{Fe}}_{3.90}{\mathrm{Mo}}_{0.08}$ microwire.

We measured the hysteresis cycles of both as-prepared samples with the help of a PPMS device, obtaining the results shown in Figure 3, while the rest of the measurements related to the hysteresis loops were taken using the fluxmetric method previously described. In both of the insets presented in Figure 3, two squared loops can be observed at low magnetic fields, with coercive field values of $H_{c}=90$ A/m and $H_{c}=40$ A/m for the Fe-rich and Co-rich microwires, respectively. This is consistent with the expected $\lambda_{s}>0$ for both microwires, although the low-field cycle of Figure 3b presents a more inclined hysteresis loop, a typical property of $\lambda_{s}∼0$ microwires.

The density was measured by taking the volume of the metallic core. This way, we estimated the magnetization saturation of both microwires, obtaining $M_{s}=850$ $\mathrm{emu}\cdot {\mathrm{cm}}^{-3}$ for the Fe-rich microwire and $M_{s}=220$ $\mathrm{emu}\cdot {\mathrm{cm}}^{-3}$ for the Co-rich one. These values were taken as a reference for the calculation of $\lambda_{s}$ for both microwires, as they are consistent with the results reported for similar Co-rich [17] and Fe-rich [18] alloys.

**Figure 4:**
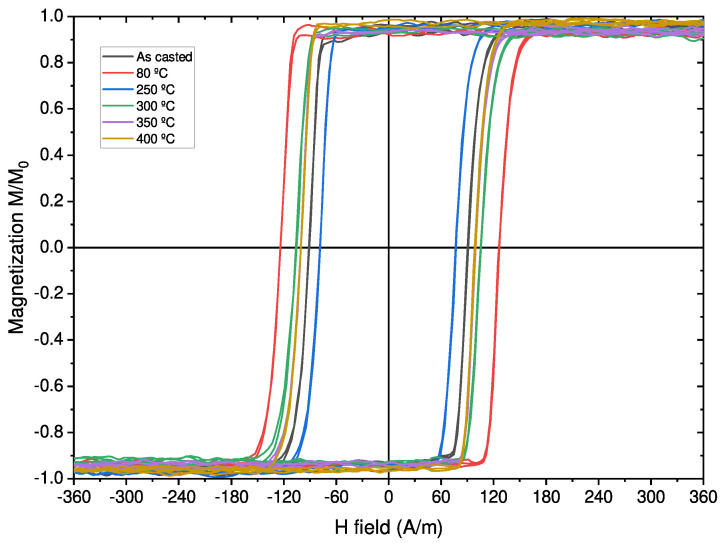
Hysteresis loops after 60 min of annealing at different temperatures with zero applied stress for the $Fe_{71.80}B_{13.27}Si_{11.02}Nb_{2.99}Ni_{0.92}$ microwire.

**Figure 5:**
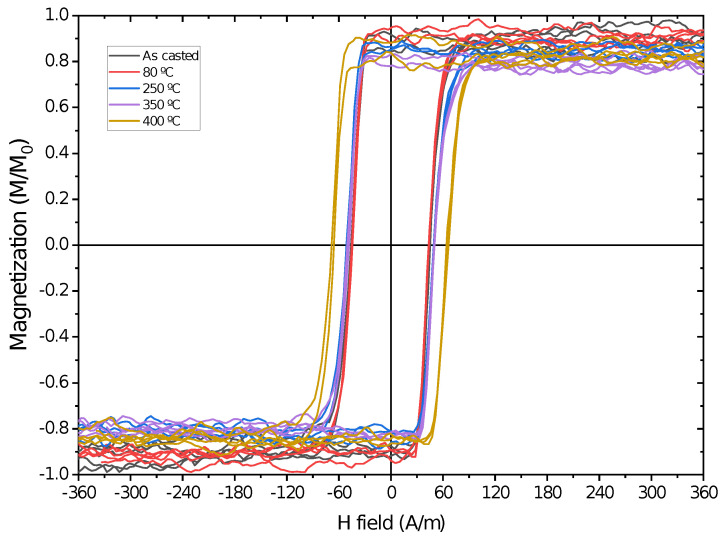
Hysteresis loops after 60 min of annealing at different temperatures with zero applied stress for the $Co_{65.34}Si_{12.00}B_{10.20}Cr_{8.48}Fe_{3.90}Mo_{0.08}$ microwire.

By measuring the magnetization at low magnetic fields, we were able to observe the effect of the annealing on the microwire’s hysteresis loops, as shown in Figure 4 and Figure 5. Values of the magnetization were normalized to the highest saturation magnetization of the measured samples in order to better compare all the samples. For both microwires, we observed a rectangular hysteresis loop with a sharp Barkhausen jump that is conserved for all annealing procedures. The rectangular hysteresis loop observed in both studied microwires is commonly attributed to the axial magnetic anisotropy of microwires with positive $\lambda_{s}$ values [1]. Such rectangular hysteresis loops of amorphous microwires are commonly explained in terms of the core–shell domain structure model, in which the domain structure of microwires is described as consisting of an inner axially magnetized single domain surrounded by an outer domain shell with radial magnetization orientation [1].

Although both microwires present rectangular-shaped hysteresis loops, we can observe that the Fe-rich Barkhausen jump in Figure 4 presents a higher $H_{c}$ value than the Co-rich microwire shown in Figure 5, indicating harder magnetic behavior for the Fe-rich sample, as also shown in Figure 3. The main effect observed in the hysteresis loops after the annealing processes is the change in the coercive field of both microwires, as the saturation magnetization remains stable in both samples. In the Co-rich sample case, the fluctuations observed in the magnetization outside the Barkhausen jumps are from the noise associated with low signal measurements.

The evolution of the coercive field in the Fe-rich microwire (Figure 4) presents irregular behavior, obtaining a maximum of $H_{c}=124$ A/m after 80 °C annealing and a minimum of $H_{c}=78$ A/m at 250 °C. This increases again at 300 °C to $H_{c}=107$ A/m and stabilizes at 350 °C and 400 °C at $H_{c}=101$ A/m. These changes in the hysteresis loops must be related to the reordering of internal stresses due to the internal stress redistribution and the change in the local atomic environment during the annealing procedures. These reorganizations and relaxations must be quite complex, as there is not an obvious relation between the abrupt changes in the coercive field evolution and annealing temperature.

The loops presented in Figure 5 show low coercive fields, with $H_{c}<100$ A/m in all the analyzed samples. Without applied stress, the coercive field remains stable up to 350 °C, while at 400 °C annealing, it increases from $H_{c}∼45$ A/m to Hc=67 A/m. This behavior points to stress relaxation due to the annealing process being capable of inducing magnetic hardening of the microwire.

**Figure 6:**
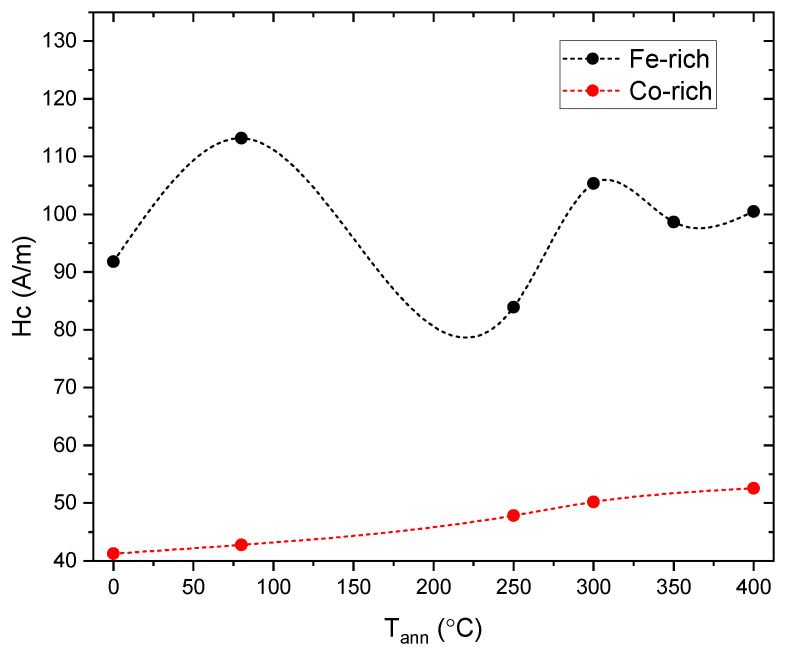
Effect of annealing temperature on coercivity of the studied microwires. The plotted dashed lines are only a visual guideline.

The evolution of coercivity versus Tann for both samples is shown in Figure 6.

**Figure 7:**
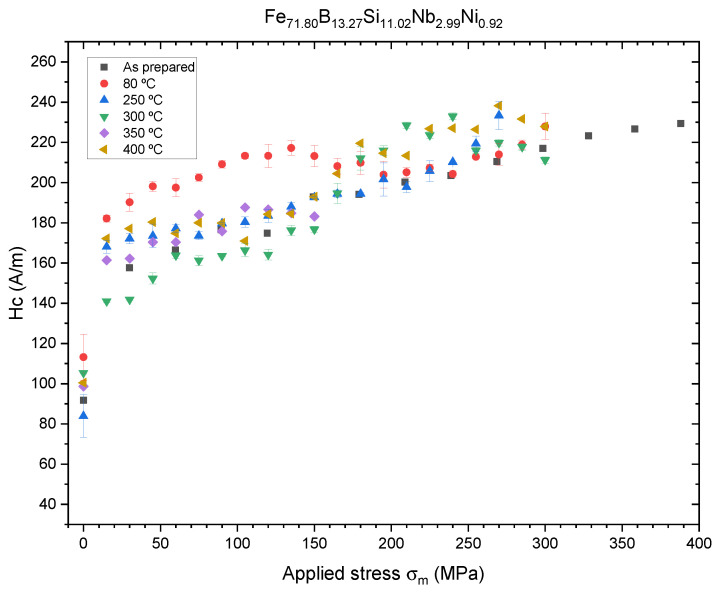
Coercitive field response to applied stress, $\sigma_{m}$, for the $Fe_{71.80}B_{13.27}Si_{11.02}Nb_{2.99}Ni_{0.92}$ microwire after 60 min of temperature annealing.

In Figure 7, we can observe the evolution of the coercitive field with applied stress for each of the annealed and as-prepared samples for the Fe-rich microwires. The results obtained are consistent with $\lambda_{s}>0$ associated with this kind of material. We observe a monotonic and convergent increase in the coercive field for all samples except the sample annealed at $T_{ann}=80$ °C. In this case, we observe a maximum in the coercive field for an applied stress of $\sigma_{m}=135$ MPa at $H_{c}=217$ A/m, which decreases afterward until converging with the rest of the samples. The observed, $H_{c}\left(\sigma_{m}\right)$, dependencies are generally similar to those previously reported for as-prepared Fe-rich microwires [9]. A more detailed discussion of $H_{c}\left(\sigma_{m}\right)$ dependencies will be provided below.

**Figure 8:**
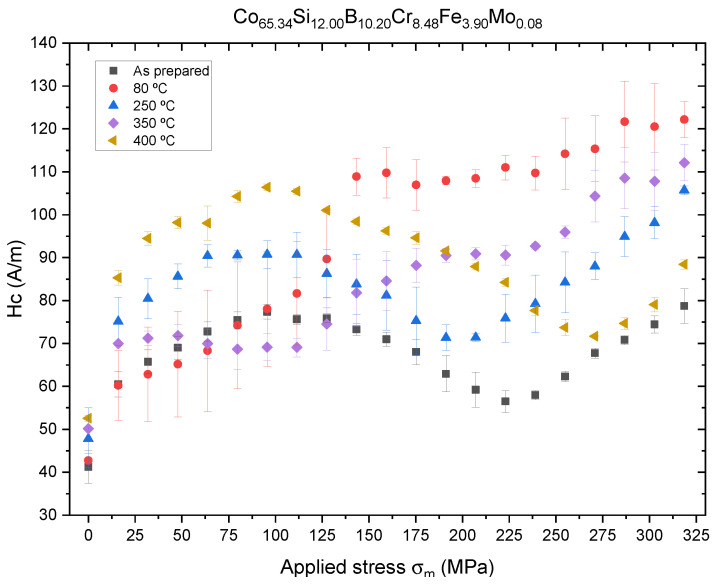
Coercive field response to applied stress $\sigma_{m}$ for the $Co_{65.34}Si_{12.00}B_{10.20}Cr_{8.48}Fe_{3.90}Mo_{0.08}$ microwires after 60 min of temperature annealing.

In the case of the Co-rich microwire, we observe a completely different behavior for the $H_{c}$ vs. $\sigma_{m}$ curve, as shown in Figure 8. At applied stresses of $\sigma_{m}∼100$ MPa, a maximum is observed coercive field values of $H_{c}=75$, 90 and 105 A/m for the as-prepared, $T_{ann}=250$ °C and $T_{ann}=400$ °C annealed samples, respectively. In the case of $T_{ann}=80$ °C and $T_{ann}=350$ °C, a monotonic increase is observed.

This behavior can be explained by supposing a $\lambda_{s}∼0$ property of Co-rich microwires with some Fe in their composition, like the one studied in this work. In this kind of microwire, it has been reported that magnetostriction, $\lambda_{s}$, follows the law given in (5):

(5) $$\lambda_{s}\left(\sigma \right)=\lambda_{s}\left(0\right)-A\sigma$$

where $\lambda_{s}\left(0\right)$ is the magnetostriction coefficient at zero applied stress and *A* is an experimentally obtained coefficient in the order of $10^{-10}$ MPa^−1^ [19] changing it to $\lambda_{s}<0$ after an applied stress threshold, as shown in [20,21]. This behavior must be associated with the redistribution of magnetic anisotropies within the magnetic core due to stress dependence of $\lambda_{s}$ and structural relaxation. Thus, it was recently observed that in the case of glass-coated microwire stress relaxation associated with annealing at elevated temperatures gives rise to an increase in $\lambda_{s}$ values [22].

In order to understand better the behavior of the coercive field response with applied stress and annealing conditions, we measured the magnetostriction coefficient, $\lambda_{s}$, using SAMR. The value of the alternate current was fixed to $I_{AC}=3$ mA for the Co-rich sample and $I_{AC}=10$ mA for the Fe-rich measurements in order to ensure reproducibility and consistency between measurements. The higher current in the Fe-rich samples was selected to compensate for the low signal recorded, which is typical for this family of alloys, while preventing the sample from suffering annealing during the measuring process due to the Joule effect [23]. The loads that induced stress, $\sigma_{m}$, on the microwires for the SAMR measurements were fixed to m=0.42, 1.03, 2.15 and 4.84 g for all samples.

**Figure 9:**
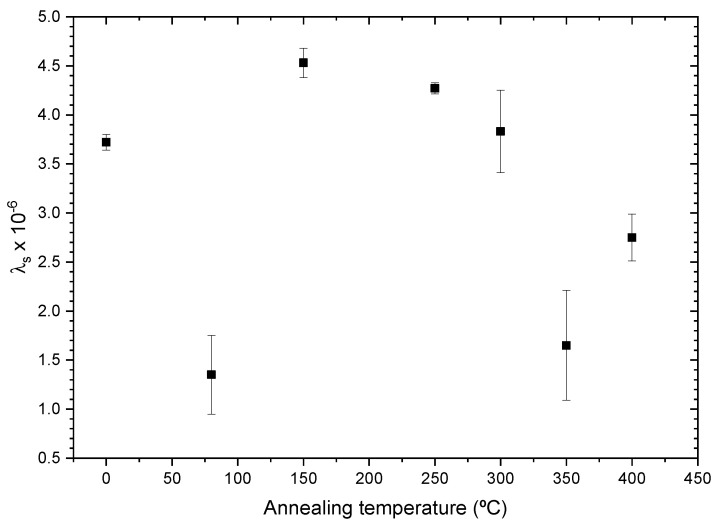
$Fe_{71.80}B_{13.27}Si_{11.02}Nb_{2.99}Ni_{0.92}$ microwire magnetostriction constant evolution with annealing temperature.

In Figure 9, we can observe the evolution of the magnetostriction coefficient with the annealing temperature for the Fe-rich alloy. All samples present high values in the order of $\lambda_{s}∼10^{-6}$, which explains the similar evolution for all samples of $H_{c}$ with the applied stress observed in Figure 7. It is worth noting that the obtained $\lambda_{s}$ values are lower than the values reported for Finemet-type amorphous ribbons [24]. Such differences can be related to either slightly different chemical compositions or the influence of internal stresses induced by the glass coating. A sharp reduction in $\lambda_{s}$ is observed from $3.72\pm 0.08\times 10^{-6}$ to $1.35\pm 0.40\times 10^{-6}$ at 80 °C, which correlates with an increase in the $H_{c}$ value and sensibility related to the $H_{c}$ vs. $\sigma_{m}$ curve. This increase in sensibility could be exploited for the fabrication of sensors with an increased sensibility, as the curve maintains the same shape as reported in our previous work [8] and could be interpolated in the same way up to $\sigma_{m}=150$ MPa. At $T_{ann}=150$ °C, the magnetostriction coefficient presents a maximum, $\lambda_{s}=4.53\pm 0.15\times 10^{-6}$, and then starts to decrease to values lower than the as-prepared sample.

**Figure 10:**
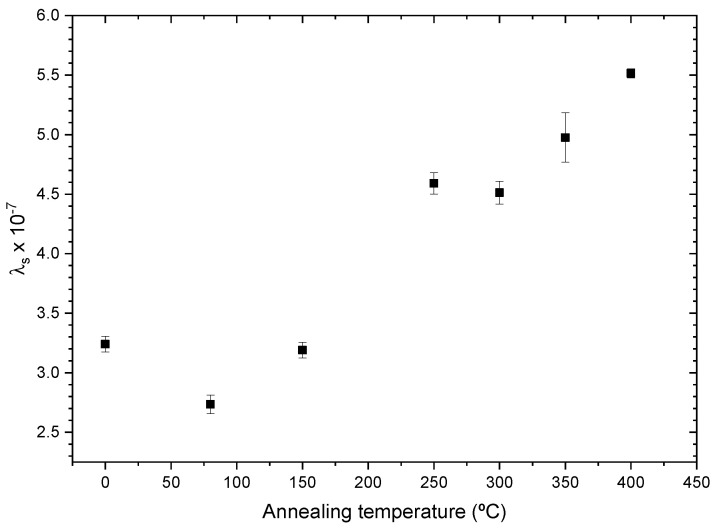
$Co_{65.34}Si_{12.00}B_{10.20}Cr_{8.48}Fe_{3.90}Mo_{0.08}$ microwire magnetostriction constant evolution with annealing temperature.

In the case of the Co-rich alloy, we observe a low magnetostriction coefficient for all annealing procedures, $\lambda_{s}∼10^{-7}$, as shown in Figure 10. In this case, we observe a magnetic hardening with an increase in annealing temperature, where the sample passes from $\lambda_{s}=3.24\pm 0.07\times 10^{-7}$ for the as-prepared sample to $\lambda_{s}=5.52\pm 0.04\times 10^{-7}$ at $T_{ann}=400$ °C. An annealing process of 1 hour at $T_{ann}=400$ °C seems to be optimal for structural health monitorization devices as the higher $\lambda_{s}$ values ensure higher magnetic stability and sensibility of hysteresis loops to applied stress.

However, the monotic increase in the magnetostriction coefficient cannot explain, by itself, the anomalous behavior of the coercive field with applied stress observed in Figure 8. This is due to the fact that our SAMR set-up only allows the measurement of $\lambda_{s}$ in a stationary regime and not while the sample is suffering stress, preventing us from establishing a relationship, like the expression given in (5), or relating the changes to a reordering of internal stresses and magnetic anisotropy in the Co-rich microwire.

## 4. Discussion

In both cases, we observe a sharp decrease in the magnetostriction coefficient after the 80 °C annealing. This change can only be attributed to the complex processes upon annealing of amorphous alloys involving internal stress relaxation together with short-range atomic ordering, structural relaxation, pair ordering and clustering, which can also affect the coercivity [25,26,27,28]. Such changes in short-range atomic ordering can substantially affect magnetic properties, such as the coercivity and magnetostriction coefficient of amorphous alloys [25,26,27].

At higher temperatures, we observe a softer change in $\lambda_{s}$, as some mechanisms of short-range atomic ordering, like pair atomic ordering, cannot be observed above Curie temperature [26].

We have studied the effect of annealing temperature and applied stress on the magnetic properties of $Fe_{71.80}B_{13.27}Si_{11.02}Nb_{2.99}Ni_{0.92}$ (d=15.9 μm and D=24.5 μm) and $Co_{65.34}Si_{12.00}B_{10.20}Cr_{8.48}Fe_{3.90}Mo_{0.08}$ (d=17.5 μm and D=22.2 μm) microwires over a wide range of temperatures and observed relevant changes in the coercitive field associated with the complex behavior of studied microwires related to the evolution of various properties, such as the magnetostriction coefficient, $\lambda_{s}$.

Although the applied stress plays a relevant role in the behavior of the hysteresis loops, short-range atomic ordering and internal stress relaxation upon annealing and of the microwire seem to be relevant. The effect of conventional and current annealing has been previously studied in soft magnetic wires and ribbons [17,18,19,20,21,22,23,24,25,26], showing nontrivial relations with the magnetostriction coefficient. The internal stress relaxation is usually associated with a decrease in Hc values during annealing [1]. However, in amorphous alloys containing more than one ferromagnetic element (i.e., Fe-Ni- and Fe-Co-based amorphous alloys), a substantial magnetic hardening after annealing is often reported [12,26,29,30,31]. The origin of such magnetic hardening of amorphous materials upon annealing is commonly attributed to the directional ordering of atomic pairs or compositional and topological short-range ordering, as well as the evolution of $\lambda_{s}$ upon annealing [28,29,30,31]. Accordingly, the unexpected increase in the $H_{c}$ value upon annealing (see Figure 4 and Figure 5) can be explained by considering directional atomic pair ordering mechanism as well as topological and compositional short-range atomic ordering and clustering. Indeed, magnetostriction is influenced by local atomic environments, the presence of clusters and even stresses [21,22]. In the present case, the complex evolution of $\lambda_{s}$ upon annealing (see Figure 7 and Figure 8) confirms the contribution of the structural relaxation of the local atomic structure. Therefore, both internal stress relaxation and structural (topological or compositional) relaxation upon annealing must be taken into account for the interpretation of $H_{c}$ evolution upon annealing and applied stresses.

Further study will be needed in order to obtain more information about the behavior, $\lambda_{s}$, of the $Co_{65.34}Si_{12.00}B_{10.20}Cr_{8.48}Fe_{3.90}Mo_{0.08}$ microwire. The main peculiarity of the studied microwires is related to the elevated value of internal stresses [3,4,32]. The largest internal stresses of preferentially axial origin are induced by the difference in the thermal expansion coefficients of the metallic alloy and glass coating [3,4,32].

As discussed elsewhere [33], the radius of the inner axially magnetized core, $R_{c}$, can be estimated from the squareness ratio, $M_{r}/M_{s}$, as:

(6) $$R_{c}=R{\left(M_{r}/M_{s}\right)}^{\left(1/2\right)}$$

where *R* is the microwire radius.

However, as can be observed from Figure 4 and Figure 5, in the studied microwires, $M_{r}/M_{s}=1$. Consequently, we must assume that due to the strong internal stresses of axial origin, the volume of the inner domain is close to the whole volume of the metallic nucleus. Previously similar experimental results have been reported [34]. Previously, $H_{c}\left(\sigma_{m}\right)$ dependence was interpreted by considering that $H_{c}$ is proportional to the energy required to form the domain wall, which is involved in the bistable magnetization process [9,35]. The domain wall energy related to the magnetoelastic anisotropy is given as:

(7) $$H_{c}\propto \gamma \propto \frac{\left[\;\frac{3}{2}A\lambda_{s}\left(\sigma_{m}+\sigma_{i}\right)\right]{\;}^{1/2}}{cos\alpha }$$

where α is the angle between magnetization and the axial direction, *A* is the exchange energy constant and $\sigma_{i}$ is the internal stress. Consequently, $H_{c}$ must be proportional to $\sigma_{m}^{1/2}$ for $sigma_{m}>\sigma_{i}$ and cosα=1. However, internal stress relaxation upon annealing takes place. Therefore, in annealed samples, $H_{c}\left(\sigma_{m}\right)$ dependence becomes more complex. The relaxation of such internal stresses can result in the redistribution of internal stresses and can substantially affect the applied stress sensitivity of glass-coated microwires, making them more suitable for applications in magnetoelastic sensors [9].

Further experiments should conducted measuring the value of $\lambda_{s}$ while applying stress. This could help in the understanding of the mechanisms behind the anomalous behavior of the Co-rich microwire and the effect of temperature annealing on the magnetic anisotropy distribution of the microwire.

## 5. Conclusions

The effect of annealing temperature and applied stress on the magnetic properties of $Fe_{71.80}B_{13.27}Si_{11.02}Nb_{2.99}Ni_{0.92}$ and $Co_{65.34}Si_{12.00}B_{10.20}Cr_{8.48}Fe_{3.90}Mo_{0.08}$ microwires prepared using the Taylor–Ulitovsky method was studied. Annealing allows tuning the stress dependence of the coercive field, indicating nontrivial changes in the microwire magnetic anisotropy. The effect of applied stimuli on the magnetic anisotropy and magnetostriction coefficient in both microwires was also discussed. For the interpretation of the observed dependencies, the stress dependence of the magnetostriction coefficient and structural relaxation upon annealing were considered.

## Data Availability

The data presented in this study are available upon request from the corresponding author. The data are not publicly available due to data being partially restricted under project funding conditions.

## Sample Availability

Samples of the microwires ${\mathrm{Fe}}_{71.80}B_{13.27}{\mathrm{Si}}_{11.02}{\mathrm{Nb}}_{2.99}{\mathrm{Ni}}_{0.92}$ (d=15.9 μm and D=24.5 μm) and ${\mathrm{Co}}_{65.34}{\mathrm{Si}}_{12.00}B_{10.20}{\mathrm{Cr}}_{8.48}{\mathrm{Fe}}_{3.90}{\mathrm{Mo}}_{0.08}$ (d=17.5 μm and D=22.2 μm) are available from the authors.

## Abbreviations

The following abbreviations are used in this manuscript:

GMI	Giant magnetoimpedance effect
SAMR	Small-angle magnetization rotation
AC	Alternate current
